# Chromosomal Replication Complexity: A Novel DNA Metrics and Genome Instability Factor

**DOI:** 10.1371/journal.pgen.1006229

**Published:** 2016-10-06

**Authors:** Andrei Kuzminov

**Affiliations:** Department of Microbiology, University of Illinois at Urbana-Champaign, Urbana, Illinois, United States of America; University of Wisconsin-Madison, UNITED STATES

## Abstract

As the ratio of the copy number of the most replicated to the unreplicated regions in the same chromosome, the definition of chromosomal replication complexity (CRC) appears to leave little room for variation, being either two during S-phase or one otherwise. However, bacteria dividing faster than they replicate their chromosome spike CRC to four and even eight. A recent experimental inquiry about the limits of CRC in *Escherichia coli* revealed two major reasons to avoid elevating it further: (i) increased chromosomal fragmentation and (ii) complications with subsequent double-strand break repair. Remarkably, examples of stable elevated CRC in eukaryotic chromosomes are well known under various terms like "differential replication," "underreplication," "DNA puffs," "onion-skin replication," or "re-replication" and highlight the phenomenon of static replication fork (sRF). To accurately describe the resulting "amplification by overinitiation," I propose a new term: "replification" (subchromosomal overreplication). In both prokaryotes and eukaryotes, replification, via sRF processing, causes double-strand DNA breaks and, with their repair elevating chromosomal rearrangements, represents a novel genome instability factor. I suggest how static replication bubbles could be stabilized and speculate that some tandem duplications represent such persistent static bubbles. Moreover, I propose how static replication bubbles could be transformed into tandem duplications, double minutes, or inverted triplications. Possible experimental tests of these models are discussed.

## Limits and Dangers of Elevated Chromosomal Replication Complexity

**Fig 1:**
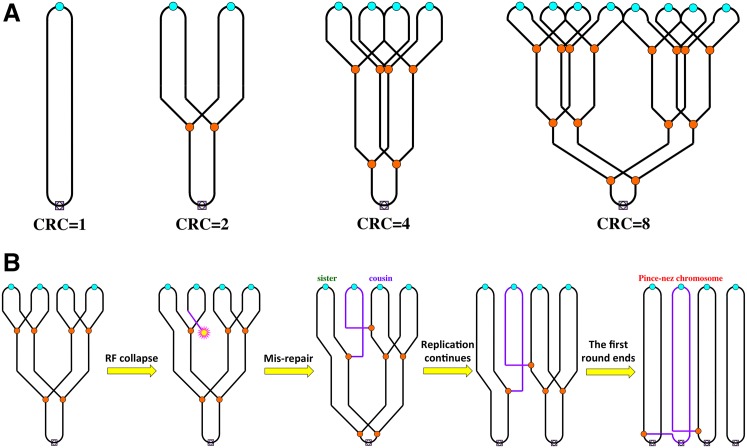
Chromosomal replication complexity: the prokaryotic perspective and the mis-repair complication. **A.** When chromosomal replication becomes rate limiting for growth, bacterial cells are capable of elevating chromosomal replication complexity up to eight. Small cyan circles denote replication origins, small orange circles denote replication forks, and small light-purple squares with an empty diamond inside denote replication termini. A nonreplicating chromosome (CRC = 1) is on the left. **B.** Recombinational mis-repair as a result of attachment of a double-strand end to a cousin (instead of the sister) DNA duplex should result in a pince-nez chromosome. Small yellow "star" marks the double-strand end formed as a result of replication fork collapse. Purple lines identify the linear chromosome linking two circular chromosomes like in pince-nez.

Chromosomal replication complexity (CRC) is defined as the ratio of the copy number of the most replicated to the unreplicated regions in the same chromosome [1]. In the eukaryotic chromosomes, with multiple and alternative replication origins firing once and only once during each cell cycle [2], CRC becomes two during S-phase and returns to one at the end of it. At the population level, replication complexity of a eukaryotic chromosome can be measured during synchronized S-phase as the ratio of the copy number of early replication origins to the copy number of chromosomal regions known to replicate late in that particular genome, like human centromeres [3] or yeast telomeres [4]. In the prokaryotic cells, with their (1) unique replication origins [5]; (2) defined termination zones [6]; and (3) cell division soon after termination of the chromosomal replication [7,8], during rapid growth with continuous replication, CRC is simply defined as the origin-to-terminus ratio [1]. Under slow growth conditions, CRC in prokaryotic cells also fluctuates between one and two (Fig 1A). However, some bacterial cells are capable of dividing two times faster than their minimal chromosomal replication time [9]. To avoid slowing their rapid growth to wait for the lagging chromosomal replication, these bacteria are capable of inducing an extra replication round in the same chromosome to bring up the trailing DNA mass synthesis rate to the cell mass increase rate and CRC to four (Fig 1A) [9–11]. The same trick also helps at moderate cell division rates when DNA synthesis is inhibited due to limited DNA precursors or a mutation in the DNA metabolism. Under these conditions, replication forks move slower, and the cells again have to induce additional replication rounds [12–15].

We have studied limits of elevated CRC in *E*. *coli* more systematically and found that when cells stabilize at CRC~8 (Fig 1A) due to modest inhibition of replication forks, they experience only modest growth inhibition. We called CRC~8 the natural CRC limit in the *E*. *coli* chromosome [1]. If replication forks are grossly inhibited, *E*. *coli* cells grow very slowly and stabilize at a much-increased CRC~22 (the functional CRC limit). Others have observed this limit before in overinitiating mutants of *E*. *coli* [16]. At both the natural and the functional CRC limits, the cell viability requires recombinational repair proficiency, suggesting formation of double-strand DNA breaks and critical need in their repair [1]. In the extreme situation in which cells have no control of a runaway initiation (achieved from an inducible replication origin), the *E*. *coli* chromosome stabilizes around an incredible CRC~64. Even though the chromosome seems to be physically intact in these cells, only one out of 20 wild-type (WT) cells survives this challenge, making it the "tolerance CRC limit" of the *E*. *coli* chromosome [1]. In contrast to WT cells, *recA* mutants survive this runaway overinitiation without loss of viability, suggesting poisoning of WT cells by recombinational repair. We hypothesized that the nature of such recombinational mis-repair, when correct repair at the DNA level generates a nonfunctional chromosome at the level of the cell, is homologous pairing in conditions of elevated CRC that leads to establishment of a new replication fork with the cousin duplex instead of the sister duplex (Fig 1B) [1]. Such a mis-repair generates a structure in which two circular chromosomes are connected by an ever-lengthening bridge of a linear third chromosome, forming the so-called pince-nez chromosome (Fig 1B) [17]—an occurrence that is currently considered lethal—as, in fact, would be any circular chromosome with an odd number of replication forks [1,18].

## Differential Replication

Are eukaryotic cells capable of elevating their CRC above two? The textbook answer to this question is "no," as the notoriously strict eukaryotic cell cycle, via the elaborate initiation control system, allows for one and only one firing event at all the replication origins licensed to fire in a given replication round [19,20]. After initiation, the spent replication initiation factors are disassembled and expelled from the nucleus into the cytoplasm, where the critical parts of the initiation machinery are degraded [21].

**Fig 2:**
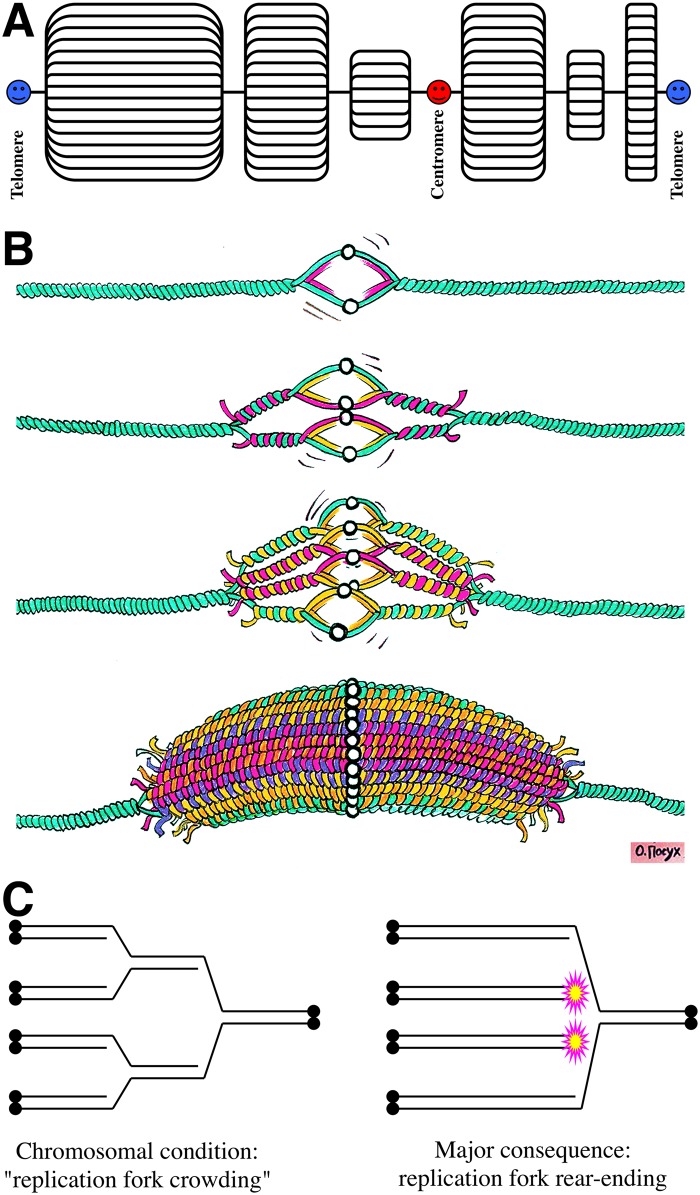
Chromosomal replication complexity: the eukaryotic perspective and replication fork rear-ending. **A.** A model of polytene chromosome of Charles Laird [26,27]. **B.** Stages of formation of an overreplicon (DNA puff) as a result of overinitiation from an unregulated replication origin in the chromosome, with a limited progress of replication forks that massively rear-end into static forks (sRFs) of the previous round. Image credit: Olga Posukh. **C.** The model of replication fork rear-ending. Double black circles denote telomeres. For clarity, a replication round consists of a single left-to-right fork. Magenta/yellow stars denote the generated double-strand ends.

Yet, examples of the so-called "differential replication" [22,23] in the cells of higher eukaryotes show that relaxation of the strict regulation of replication initiation to achieve elevated CRC in eukaryotic chromosomes is not only possible but is not unusual. Perhaps the best-known example of the grossly elevated and variable CRC on the chromosomal scale are the polytene chromosomes in higher animals and plants [24], in which centromeres and telomeres, as well as many heterochromatic regions, appear to stay single copy due to specific protein factors [25], whereas the coding regions along the chromosome are present in the highly elevated (up to a few thousand) and variable numbers (Fig 2A) [26,27]. A particular polytene chromosome phenomenon, called "splitting" [24], visually confirms variation of CRC along the chromosome length.

Sometimes an additional local overreplication amplifies only a few specific genes within a polytene chromosome. These so-called "DNA puffs" (as opposed to a more common transcription puffs) [24], or localized nested replication bubbles (Fig 2B), are observed during developmental transitions in Diptera [22,28,29] and are also called "amplicons" there [30]. (Parenthetically, the term "amplicon" in reference to the elevated copy number at DNA puffs is potentially confusing, as the first and still predominant use of "amplicon" is to describe linear tandem amplification by rolling-circle replication of short DNA segments during packaging into HSV-1 based vectors [31]. I propose to call DNA puffs "overreplicons" (Fig 2B) to stress the local nature of overinitiation in this case.) Examples of DNA puffs include *Drosophila* chorion genes, amplified in ovarian follicle cells [32,33], and salivary gland DNA puff gene in *Bradysia* [34,35]. The maximal (local) CRC reaches ~64, with more typical ranges around 16 [30]. DNA puffs are also observed in plants [36].

## Underreplication

Apparently, differential replication (both local in DNA puffs or global in polytene chromosomes) serves the purpose of maximizing gene expression [22,30]. DNA puffs maximize the output of specific genes in highly specialized cells, whereas polytene chromosomes, in addition to boosting metabolism, allow cells of certain tissues to grow big (for example, when cell-to-cell junctions are to be avoided in this location) by increasing their ploidy [37]. In both cases, differential replication is critical for the cell function and is, apparently, controlled and maintained by yet-to-be-characterized systems.

Polytene chromosomes of Diptera provide a remarkably visual example of polyploidy, but their unique feature is chromosomal condensation rather than polyploidy itself. In fact, polyploidy due to endoreduplication is widespread in differentiated cells of higher eukaryotes [38], supporting a higher metabolism and/or bigger cell volume. However, unless there are multiple nuclei in the same cell, polyploidy is not evident, because, in most cases, polyploid nuclei do not condense their chromosomes. There are at least two types of the modified cell cycle that generate polyploid nuclei: endocycle (→S→G→) and endomitosis (→G1→S→G2→ (m) →) [37]. The best-known examples in mammals for endocycle are trophoblast giant cells [39], whereas for endomitosis, these are megakaryocytes [40]. Remarkably, in contrast to the polytene chromosomes of *Drosophila* that retain the basal copy number of the heterochromatic regions [41], the two examples of the mammalian polyploid cells have uniform copy number profiles [42], with only moderate underrepresentation in the copy number of the heterochromatic relative to euchromatic regions [43].

Comparison with the polyploid nuclei makes it obvious that polytene chromosomes underreplicate their heterochromatic regions rather than overreplicate their euchromatic regions. This underreplication does not affect their elevated CRC status, but it does shift attention from the mechanisms of overinitiation at the origins to the mechanisms that suppress replication of heterochromatin and to the possible structure of a static replication fork (sRF) (Fig 2B) and the expected chromosomal lesions (Fig 2C), which will be discussed later. At least two phenomena contribute to heterochromatin underreplication at the genome level [41]: (1) active suppression of the replication initiation in heterochromatin and (2) replication fork stalling at the heterochromatin boundaries. The protein complex responsible for sRFs at the heterochromatin boundaries in *Drosophila*, whose name "suppressor of underreplication" (SuUR) reflects the phenotype of the corresponding mutant [44,45], regulates heterochromatin-specific histone modification [46]. Thus, "underreplication" is another code name for elevated CRC.

## The Onion-Skin Replication

It is remarkable how essentially the same phenomenon is known by different names in different fields. If similar local overinitiation-driven DNA puffs (Fig 2B) are induced in the chromosomes by insertion of mobile genetic elements like viruses or relaxed-copy-number plasmids, this is historically referred to as "onion-skin replication" [47]. Still, "onion-skin replication" is just a visual description of an overreplicon, so it is encouraging to see this term applied to describe the developmental DNA puffs in Diptera as well [30,48]. The important difference from the DNA puffs or polytene chromosomes above is that, because mobile elements insert at random locations of the host chromosomes, no specialized system to maintain and control stable elevated CRC is suspected in the case of overreplication from exogenous replicons.

A classic example of the onion-skin replication, the local overreplication-based DNA amplification from an exogenous origin, is observed in cells infected with polyoma viruses (like SV40) [49]. These viruses insert their genomes into the chromosome and stay dormant. Upon DNA-damaging treatment, the virus awakens before excision and induces several rounds of unscheduled replication to bring up the copy number of their genomes to over ten [49]. Another more sinister example is occasional chromosomal integration of a papilloma virus genome, which is supposed to stay as an extrachromosomal circular plasmid with an elevated copy number [50]. Naturally, the integrated papilloma virus genome tries to maintain its elevated copy number within the chromosome, inducing onion-skin replication and amplifying neighboring chromosomal regions [51]. Not surprisingly, such chromosomal integrations of papilloma virus genome frequently lead to cancer [52].

Similar events are registered in bacterial chromosomes, in which the resident prophages may undergo lytic induction preceding their excision from the chromosome [53,54] or when a plasmid with relaxed copy number inserts into the chromosome by homology [55–57]. In case of the temperate phage inducing this so-called "escape replication" [53], the cell is doomed, whereas plasmid's attempt to maintain its regular copy number within the chromosome is tolerated if this copy number is down-regulated (by suppressor mutations) but becomes problematic when the copy number reaches around 50 [57], confirming the existence of the "tolerance limit" of CRC in *E*. *coli*'s chromosome [1].

## Subreplication?

If the steady-state CRC>>2 situations above can be rationalized in terms of overinitiation, is it possible to encounter CRC < 2 in direct measurements of replicating chromosomes? Clearly, CRC < 2 in a given replicating chromosome is theoretically impossible—by definition, it has to be at least two for any replicating DNA molecule. It is also obvious that, if measured in a population of cells with only some of them in S-phase, CRC will be less than two. But can it be measured as <2 in a population of cells when all of them are replicating their chromosomes and, if "yes," does it reflect "subreplication" (some kind of a cryptic underreplication)?

**Fig 3:**
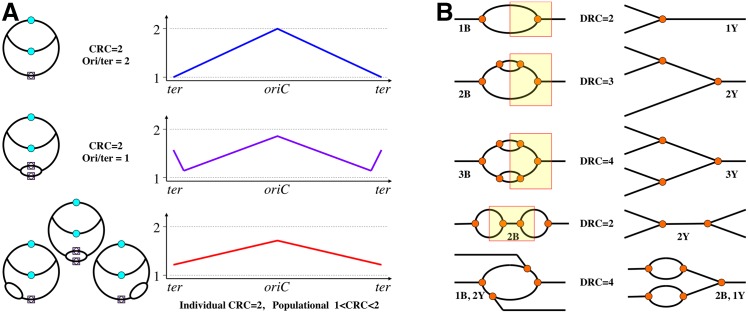
Explanation of subreplication and examples of the formalism of DNA replication complexity. **A.** Subreplication when the measurable chromosomal replication complexity is less than two. The chromosome replication schemes on the left correspond to the marker frequency profiles on the right (the chromosome is "linearized" at the terminus). The top row corresponds to WT *E*. *coli* cells, the middle row corresponds to the *recG* mutants, and the bottom row shows the *rnhA* mutants. **B.** Formalism of DNA replication complexity. DNA duplexes are represented by single lines, replication forks are marked by orange circles. Yellow rectangles on the left delineate the part of the molecule corresponding to the structure on the right. "B" stands for "bubble," and "Y" stands for a single fork. This formalism is applicable to replicating structures with a single maximum or a single minimum of replication complexity.

This question highlights the importance of the "replication-opposite" reference points for actual CRC measurement. For example, in the bacterial chromosome, with its uni-bubble format of replication, the natural reference points with opposite replication status are the replication origin and the terminus (Fig 1A). CRC in bacteria is simply expressed as the ori/ter ratio and equals two in the population in which all chromosomes have a single replication bubble (Fig 3A, top). Yet, by the same token, if there are additional initiations around the terminus in some chromosomes, the ori/ter ratio will be less than two in such a population (Fig 3A, middle). Certain bacterial mutants depart from the uni-bubble replication; in *E*. *coli*, these are *rnhA* and *recG* mutants, defective in the timely removal of R-loops [58,59]. Some of these stable R-loops spawn replication bubbles via the replication initiation mechanism used by small plasmids [60]. In addition, the *recG* mutants tend to overinitiate during double-strand break repair at D-loops [61]. Because, for unknown reasons, there is a preference for these R/D-loop initiations in the chromosomal half centered on the terminus, whereas the actual initiation positions vary from cell to cell in these cultures, the overall ori/ter ratio is significantly less than two in the *rnhA* or *recG* mutants (Fig 3A, middle and bottom) [62,63]. In fact, the R/D-loop initiations in these mutants are frequent enough to support chromosomal replication if the designated chromosomal origin, *oriC*, is deleted, with the expected inversion of the chromosomal replication profile [62–64].

It should be stressed that, in any particular chromosome in these mutants that has a single origin-initiated bubble, CRC is still strictly two (Fig 3A), because the ratio of the copy number of replicated to unreplicated regions within a single chromosome cannot be a noninteger. At the same time, at the populational level, because of the "less than one" frequency and random position of these R/D-loop initiations, the ratio of the "designated as most replicated" (*oriC*) to the "designated as least replicated" (*ter*) chromosomal regions becomes less than two, flattening the replication profiles of such chromosomes (Fig 3A, bottom) [62,65] and suggesting subreplication. In fact, just having an additional fixed-position ectopic replication origin in the *E*. *coli* chromosome already lowers population-average CRC below two [66], demonstrating another factor in reduction of the population-average CRC, which is shortening the chromosomal replication time (also observed in mutants in the nucleoid-associated proteins [67,68]).

Interestingly, the population-average CRC in the eukaryotic cells may also be less than two during the S-phase—for example, at the minor replication origins or in the chromosomal arms replicating late (or slowly) [69]. Still, if CRC is determined as the ratio of the regions that replicate early in all cells to the regions that replicate late in all cells, it is strictly two in a population of S-phase eukaryotic cells [69]. In summary, subreplication as an empirical phenomenon emphasizes various factors complicating both CRC measurements and their interpretation.

## DNA Replication Complexity

The previous discussion makes it clear that various regions in the same chromosome may have distinct replication complexities. For example, in the *E*. *coli recG* mutant, in conditions of rapid growth, the two replication rounds coming from *oriC* will be met by an additional replication bubble at the terminus region. Or, there could be regular bubbles along a eukaryotic chromosome and, among them, the onion-skin structure at the viral genome insertion site. Perhaps the most convincing illustration of the intrachromosomal variation of local CRC is the "RC-fest" of the polytene chromosomes (Fig 2A). In all these cases of "intrachromosomal differential replication," the chromosome-wide replication complexity concept loses its descriptive usefulness. The only thing that remains constant among all these examples is the replication complexity of two around any replication bubble closest to its replication origin.

All these complications illustrate the fact that the original term "chromosomal replication complexity" applies for undisturbed (WT) replication patterns of both prokaryotic chromosomes (unique origin, variable number of initiations) and of eukaryotic chromosomes (multiple origins, strictly one initiation per cell cycle). Examples of alternative origins with a variable number of initiations in the same chromosome call for metrics of the replication complexity at the subchromosomal scale. A useful term may be "local replication complexity" of a replicon, the "replicon" being defined as the DNA segment replicated from a single initiation site. Practitioners view replication complexity via the prism of methods like 2-D agarose gel electrophoresis for discrimination between various branched DNA species (Fig 3B) [70,71] and would appreciate their own term. Such detection method-friendly metrics for molecular biology could be "DNA replication complexity" (DRC) (the ratio of the copy number of the most replicated to the nonreplicated parts of a defined chromosomal segment with a single replication origin or terminus) as a characteristic of branching in any defined DNA piece, precisely describing the number of replication bubbles and individual forks in it (Fig 3B).

## Re-replication Destabilizes Chromosomes

As mentioned in the introduction, increased CRC in *E*. *coli* is linked to formation of double-strand DNA breaks [1,72–74], so cell survival becomes dependent on recombinational repair [73,75,76]. The same relationship is found in human cells, in which relaxed control over replication initiation in certain mutants results in more than one firing from some replication origins within a single replication round, leading to local overreplication (called "re-replication") [20]. Re-replication and onion-skin replication in human cell lines cause formation of double-strand DNA breaks and dependence of these cells on recombinational repair [77]. Similarly, the under-replicated heterochromatic regions in the *Drosophila* polytene chromosomes accumulate double-strand ends [78] and are sites of binding of histone gamma-H2A, the hallmark of double-strand ends [44]. Onion-skin replication in the *Drosophila* follicle cells also attracts histone gamma-H2A binding and has to be supported by double-strand break repair [32]. Thus, in both prokaryotic and eukaryotic experimental systems, elevated CRC causes chromosomal fragmentation and dependence on double-strand break repair.

The model of replication-dependent double-strand DNA breakage that explains this chromosomal fragmentation best is "replication fork rear-ending" due to replication fork crowding and sRFs (Fig 2C) [78–81]. In its essence, when there is more than one replication round in the same DNA and replication forks of the previous rounds are stalled or move slower than replication forks of the subsequent rounds, the latter may rear-end the former, releasing two of the four replication arms as double-strand ends (Fig 2C).

Replication fork rear-ending with subsequent homology-driven reassembly predicts that, in DNA with repeats, repeat-mediated rearrangements will be stimulated. Indeed, re-replication in eukaryotic cells elevates the frequency of rearrangements [82–84] and causes cancer in humans [84–86]. Thus, the three classic hallmarks of genetic instability—(1) formation of double-strand ends; (2) dependence of the affected cells on double-strand break repair; and (3) increased repeat-mediated chromosomal rearrangements—are all present in cells with elevated CRC, establishing elevated CRC as a factor of genome instability. There were several independent proposals some 30 years ago linking re-replication with genome instability [84,87–89].

## Amplification Versus Replification

**Fig 4:**
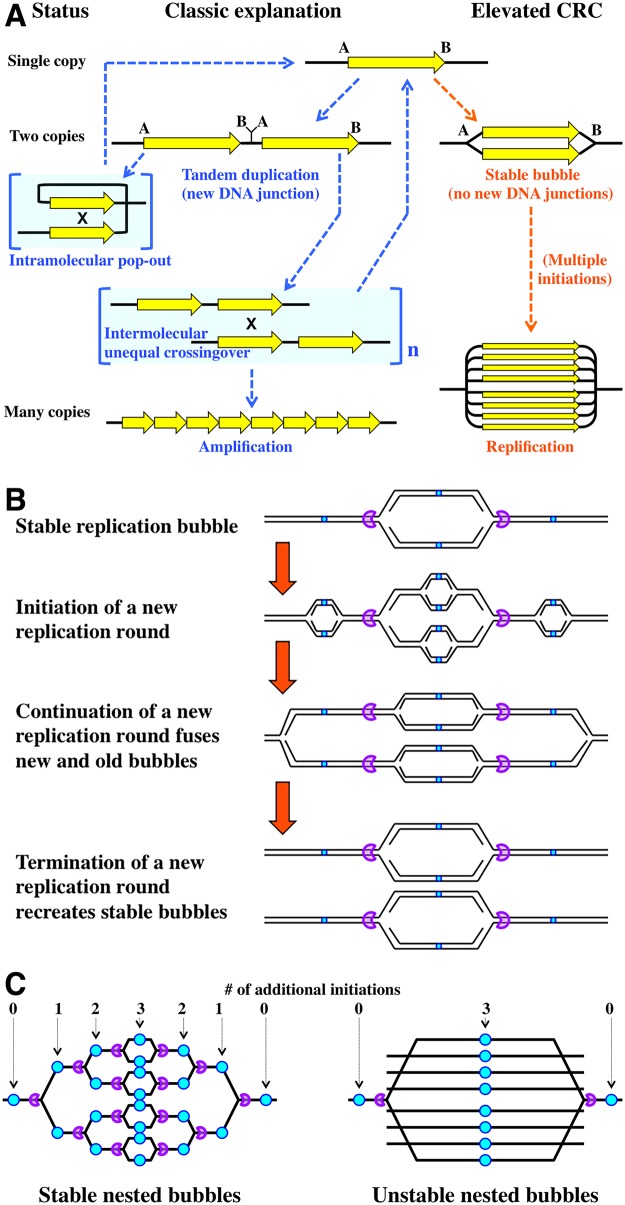
Static replication bubbles. **A.** Amplification (tandem iteration) versus replification (elevated replication complexity). The classic model of tandem duplication leading to amplification via unequal crossing-over is shown on the left. The possibility of the corresponding elevated replication complexity (replification) is shown on the right. **B.** A combination of unidirectional termination sites (purple pacman "pokemons") and appropriately spaced replication origins (tiny cyan circles) should be able to stably maintain elevated replication complexity of a chromosomal region through a replication round. **C.** Static nested bubbles require a system of alternating replication origins and unidirectional termination sites. If there is only one pair of termination sites around a single replication origin, the nested bubbles cannot be stable and disintegrate via replication fork rear-ending (Fig 2C).

Any region of a chromosome, in either prokaryotes or eukaryotes, is tandemly duplicated in a population with a frequency of 10^−3^ [89,90]. A tandemly duplicated region is "copy-number-unstable" in that it can be either further amplified, or resolved back to a single copy (Fig 4A, left), by homologous recombination via intermolecular unequal sister-chromatid exchange, as first proposed by Sturtevant [91], or via intramolecular pop-out. According to this paradigm, amplification of a chromosomal region is a two-step process: the slow formation of a "founder" tandem duplication is followed by a much faster amplification to multiple copies or reversal to a single copy (Fig 4A, left). Alternatively, there are also schemes that envision amplification as a single multistage catastrophic event [92], notably, the "spiral amplification" idea [93].

I have noticed that the elevated CRC situation offers a 2-D alternative to the classic linear duplication/amplification scheme. Indeed, some of these duplications and amplifications could be in fact regions of stably elevated CRC—basically, static replication bubbles or sets of nested static bubbles (Fig 4A, right). To stress its replicative nature, I propose the term "replification" for such amplification by localized overinitiation, in contrast to the classic "amplification" by tandem iteration. In nondividing cells, such static replication bubbles could be stabilized by replication fork "locking" [94,95]. In cycling cells, static bubbles could be facilitated by pairs of unidirectional termination sites (Fig 4B) and by analogy with such termination sites in the *E*. *coli* chromosome and the RFB sites in the eukaryotic rRNA operons [6]. If replication from the "outside" replication origins reaches these termination sites before replication from the "inside origin," the new replication round across the preexisting bubble will simply duplicate it (Fig 4B). An illustration of this scenario is found in the *E*. *coli recG* mutants, in which replication bubbles robustly initiate both at *oriC* and in the terminus, but progress of the terminus bubble is soon constrained by the termination sites, leading to the characteristic bi-modal chromosomal marker frequency profile (Fig 3A middle) [63,65,96].

The robust scenario of a static bubble can be scaled up to explain a frozen set of nested bubbles (replification), with each bubble blocked at its dedicated pair of termination sites as long as there are replication origins between termination sites (Fig 4C, left). With these alternating origins and termination sites (the arrangement found, for example, at the eukaryotic ribosomal DNA array [6]), such replification structure could become quite complex, maintaining the desired copy number of the region (Fig 4C, left). As an interesting, simpler variation of this arrangement, if there are multiple firings of a replication origin between the closest pair of termination sites, the replified structure becomes unstable in this case, as multiple replication forks rear-end into the original forks blocked at the termination sites, forming linear DNA fragments spanning the chromosomal segment between the termination sites (Fig 4C, right, a schematic presentation of Fig 2B). The scenario analogous to unstable nested bubbles is observed in the underreplicated heterochromatic regions of polytene chromosomes [25,46,78].

## Conversion of a Static Replication Bubble into a Tandem Duplication

Although the replification scenario looks mechanistically sound, its pure form explains only amplifications with no new DNA junctions, whereas a lot of amplifications are known to be associated with new DNA junctions ("B/A" in Fig 4A). In fact, these novel DNA junctions associated with tandem duplications caused initial attention because of the expected insights into the mechanisms of formation of the founder tandem duplications. However, in eukaryotes, these junctions were invariably found to have either no homology or a microhomology of one-to-few nucleotides between the joined ends [92,97]. In bacteria, the level of microhomology at the new junctions tends to be higher ([98], reviewed in [99]), and these rearrangements are more frequent in mutants with replication defects, inspiring models based on various long-range template switching events at stalled or broken replication forks [100–102]. Formation of the novel DNA junctions could have been mechanistically independent of amplification, but, at least in some cases, specific new DNA junctions were amplified with the rest of the amplified DNA segment, meaning that formation of the junction must have preceded amplification.

**Fig 5:**
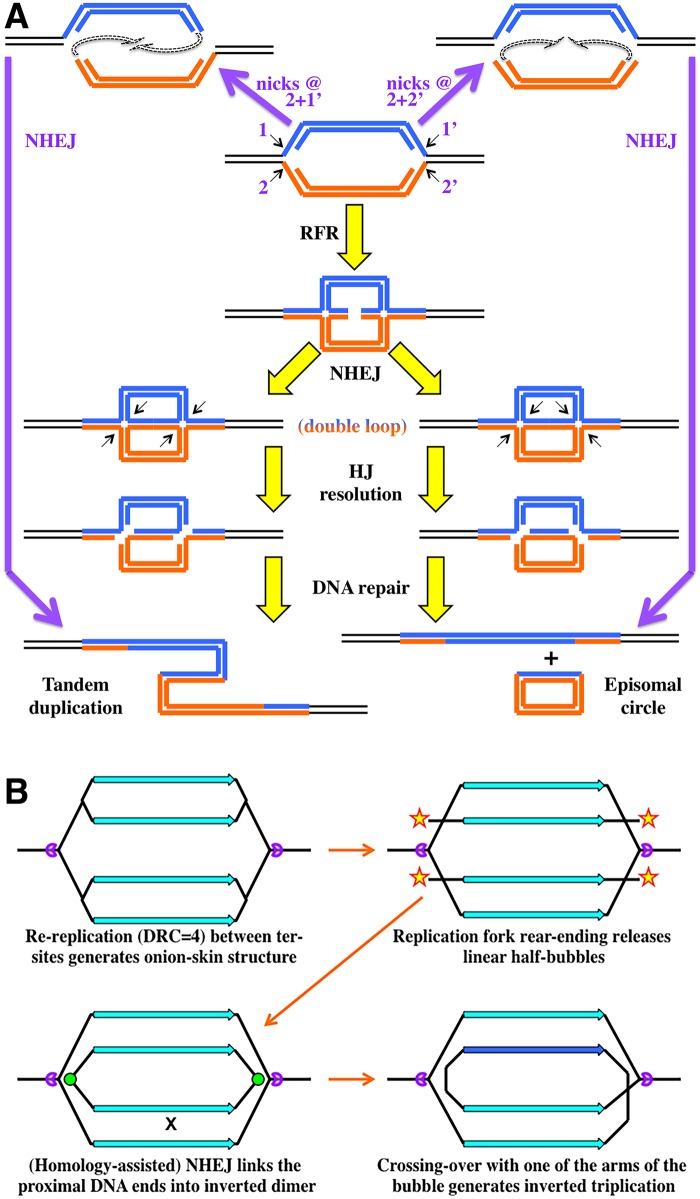
Models of a static replication bubble conversion into chromosomal rearrangements. **A.** A possible conversion of a static replication bubble into a tandem duplication or a popped-out circle by either a combination of nicks at replication forks and NHEJ (purple arrows) or a combination of replication fork reversal and NHEJ (yellow arrows). The sister arms of the bubble are marked either blue or orange to facilitate recognition of their DNA strands. HJ, Holliday junction. Small arrows, nicks. **B.** A model of how inverted triplications may form via replication fork rear-ending with subsequent NHEJ and crossing-over. Purple "packmen," directional ter-sites; yellow stars, double-strand ends; green circles, new DNA junctions by NHEJ.

Several general schemes explaining formation of the initial tandem duplication have been proposed by the mid-1980s, some of them featuring replication bubble intermediates [87–89], but they understandably lacked mechanistic details (thus, predictive power), because two important phenomena of the DNA metabolism—the existence and processing of sRFs (in particular, replication fork regression [RFR]) [103–105] and the nonhomologous end joining (NHEJ) [106]—were discovered a full decade later. Interestingly, NHEJ, in combination with sRF processing in general, and RFR, in particular, offer plausible scenarios to convert static replication bubbles into tandem duplications (Fig 5A, the yellow arrows). In a nutshell, after regression at both forks of a static replication bubble, the two novel double-strand ends are joined by NHEJ, while the resulting double-ring intermediate is resolved at the Holliday junctions to produce tandem duplication in half of the resolutions (Fig 5A, left). A simpler scenario, initiating with sRF nicking instead of RFR, is also possible (Fig 5A, the purple arrows).

Is there any evidence for or against this sRF processing followed by NHEJ among the duplication/amplification data? The two major predictions of the sRF-NHEJ scheme (Fig 5A) is that (1) in half of the cases, the double loop intermediate has to be resolved to pop-out an extrachromosomal circle and (2) the two resolution options are mutually exclusive, in that, from a particular static bubble, either the tandem duplication forms or the episomal circle pops out, but not both. Remarkably, an important "clarification" of the nature of the tandem duplication from cells of higher eukaryotes (which was completely lost in bacteria due to their unique chromosomal origins of replication) was realization that the amplification can be either intrachromosomal (tandem amplification proper, detected as eventual formation of "homogeneously-staining regions" [HSRs] in the chromosome, reflecting subsequent amplification) or extrachromosomal, in the form of "double-minute" (DM) circles [87,89]. Such extrachromosomal circular duplications of chromosomal segments are especially common in solid tumors, in which they amplify various chromosomal regions [107]. Remarkably, even though the same cell may carry both HSR and DM amplifications of the same DNA region as long as their novel DNA joints are different, a particular amplification with a specific DNA junction can be either HSR or DM, but never both in the same cell [87,89]. This observation that the same early amplification intermediate is resolved to give either HSR (tandem duplication) or DM (circle pop-out), but never both, matches the predictions of the sRF-NHEJ model (Fig 5A). The model is directly testable in an appropriate experimental setup.

## Inverted Triplications from Unstable Nested Bubbles

An interesting scenario of NHEJ-mediated rearrangements can be envisioned at the unstable nested bubbles (over-replicons), at which multiple linear DNA fragments are proposed to be released by rear-ending of replication forks into the static fork at the termination site (Fig 4C, right) [78,80]. In the simplest case of DRC = 4, two such linear fragments will form between the termination sites (Fig 5B). In principle, such linear products of replication fork breakage are known to occasionally circularize [108]; however, in this case when the two fragments are released simultaneously, due to their proximity and longitudinal alignment, the open duplex ends could be fused together by NHEJ (which may be even assisted by homologous pairing in this case [109]), resulting in formation of an inverted dimer circle (Fig 5B) [109]. Finally, homologous recombination of this inverted dimer circle with one of the bubble arms generates inverted triplication (Fig 5B)—a distinct and odd-looking product, but a strong prediction of the fork rear-ending scenario. This scheme is robust against a scale-up to multiple released linear fragments: in fact, the whole final amplified product can be "self-assembled" by NHEJ alone if the remaining forks regress. Remarkably, many amplifications both in bacteria and eukaryotes are in fact based on inverted duplications (that is, they started as inverted triplications) [92,99,110] rather than on tandem duplications.

Two ingenious models have been proposed recently to explain formation of inverted triplications [110,111], but because in both cases the repeated region was found bracketed by short inverted repeats, both models are based on template switching at these inverted repeats, either by primer migration from the template [112] or by replication fork locking [94]. In fact, if template switching is appropriate (when preexisting inverted repeats are found at the junctions), then the much earlier model for the formation of arrays of inverted repeats—the sophisticated idea of spiral amplification—also initiated with replication fork locking [93]. Our model of (2-D) replification to (1-D) amplification conversion (Fig 5B) is different from these template-switching-based models in that it has no requirements for short inverted repeats and, in fact, predicts lengthy spacers between the two inverted regions, derived fully from DNA sequences contiguous with one of the repeated regions (because of replication fork rear-ending at varied locations around the blocked forks), which is exactly what is found in many of these inverted amplifications [92].

## Conclusion

The novel metric—chromosomal replication complexity—spans from the typical in vivo CRC~2 in most chromosomes, past the increased CRC of the onion-skin replication and DNA puffs, to the highly amplified CRC of the polytene chromosomes. Because in some cases CRC may vary within the same chromosome (intrachromosomal differential replication), a more general metric—DNA replication complexity (DRC)—is also introduced. This metric is applicable more broadly, from pure (short) DNA molecules detectable in vitro by 2-D gels to the individual (over)replicons or underreplicated sites within chromosomes. Stable elevated CRC highlights a group of related phenomena, in which the central role is played by formation and processing of sRF, a static replication fork. I also propose that static replication bubbles might be behind some cases of apparent tandem duplications, whereas over-replicons (nested sets of static bubbles) could be the real structures behind some amplifications (assumed to be tandem iterations) (Fig 4A).

Increased CRC is a factor of genome instability, in all known cases acting not only to induce chromosomal damage but also to confound its subsequent recombinational repair. Elevated replication complexity promotes recombinational misrepair of disintegrated replication forks, as the double-strand end in the replified (locally overreplicated) portion of the chromosome can be homologously attached not only to the intact sister duplex (correct repair) but also to one of the several cousins (misrepair) (Fig 1B) [1]. The presence of DNA repeats further confuses recombinational repair, leading to gross chromosomal rearrangements. In addition, sRFs may be processed (regressed or broken), allowing NHEJ to form tandem duplications or other local rearrangements based on microhomology (Fig 5A). In fact, it is tempting to speculate that even the nucleolus-forming chromosomal region with tandem arrays of rDNA in eukaryotic cells has been converted from the initial DNA puff (over-replicon) by a combination of sRF processing-NHEJ and homologous crossing-over. DNA puffs at rDNA regions are known [113–115].

The recognized importance of the elevated CRC factor in the overall chromosomal metabolism poses new questions and opens new experimentation venues. Do bacteria possess a system to resolve pince-nez (or, more generally, sigma-replicating) chromosomes (Fig 1B)? Such a capability would be a lifesaver for prokaryotic cells. The bacterial terminus, bracketed by the inverted termination sites, is a well-known system to ensure that replication is unidirectional through most of the prokaryotic circular chromosome, but do similar developmental stage-specific systems maintain region-specific over-replicons in the polytene chromosomes of eukaryotes? The SuUR protein in *Drosophila* may be a component of one such system [25,46]. There also has to be a general system that controls spreading along the chromosome of onion-skin replication initiated from randomly inserted mobile elements.

The mechanisms of genetic instability associated with elevated CRC need to be explored. The current model of replication fork rear-ending (Fig 2C) [78–81] predicts that the overreplicon structure (Fig 2B) will be maintained by recombinational repair. However, the only study that looked into the extent of replification in DNA repair mutants found the effect of NHEJ rather than recombinational repair [32]. If confirmed, this will dramatically change the models of replification.

The possibility that some tandem duplications and higher copy number variations are in fact static replication bubbles needs to be tested by identifying the associated new junction sequences. If some of these copy number variations have no new junction sequences, especially in cases in which they are bracketed by known termination sites, static bubble explanation should be considered. There are at least two differences between tandem amplification versus replification phenomena: (1) amplicons have sharp copy number-change borders separating them from single copy sequences around, whereas over-replicons have gradual borders with apparent slopes, reflecting gradient of static nested bubbles, and 2) amplifications can have any number of copies, whereas replifications should always comprise 2^n^ copies.

The model in Figs 4 and 5 generates strong predictions: (1) if a replication origin is bracketed by a pair of inward-oriented termination sites (like in the bacterial chromosomal terminus), a static replication bubble may form as a result of occasional unscheduled initiation from the origin; (2) this origin bracketed by termination sites should be prone to tandem duplication (and subsequent amplification). Tandem duplications of the terminus region in the *recG* mutants in *E*. *coli* could be expected (if the terminus duplication is permitted in the bacterial chromosome), but bacteria generally lack active NHEJ, so these experiments are better suited for cells of higher eukaryotes.

In summary, the phenomenon of replification offers a fresh look at the chromosomal structure and dynamics via a new metric of chromosomal/DNA replication complexity by providing a systemic view on the various instances of elevated replication complexity within over-replicons and their important consequences for genome instability via formation and processing of static replication forks.
